# Biofortification of Silage Maize with Zinc, Iron and Selenium as Affected by Nitrogen Fertilization

**DOI:** 10.3390/plants10020391

**Published:** 2021-02-18

**Authors:** Djordje Grujcic, Atilla Mustafa Yazici, Yusuf Tutus, Ismail Cakmak, Bal Ram Singh

**Affiliations:** 1 Faculty of Environmental Sciences and Natural Resource Management, Norwegian University of Life Science, 1432 Ås, Norway; djgrujcic@yahoo.com; 2 Faculty of Engineering and Natural Sciences, Sabanci University, Istanbul 34956, Turkey; ayazici@sabanciuniv.edu (A.M.Y.); ytutus@sabanciuniv.edu (Y.T.); cakmak@sabanciuniv.edu (I.C.)

**Keywords:** biofortification, maize, micronutrients, nutrient uptake, plant nutrition

## Abstract

Agronomic biofortification is one of the main strategies for alleviation of micronutrient deficiencies in human populations and promoting sustainable production of food and feed. The aim of this study was to investigate the effect of nitrogen (N)fertilization on biofortification of maize crop (*Zea mays* L.) with zinc (Zn), iron (Fe) and selenium (Se) grown on a micronutrient deficient soil under greenhouse conditions. Factorial design experiment was set under greenhouse conditions. The experiment consisted of two levels of each N, Zn, Fe and Se. The levels for N were 125 and 250 mg N kg^−1^ soil; Zn were 1 and 5 mg Zn kg^−1^ soil; levels of Fe were 0 and 10 mg Fe kg^−1^ soil; levels of Se were 0 and 0.02 mg Se kg^−1^ soil. An additional experiment was also conducted to study the effect of the Zn form applied as a ZnO or ZnSO_4_ on shoot growth, shoot Zn concentration and total shoot Zn uptake per plant. Shoot Zn concentrations increased by increasing soil Zn application both with ZnSO_4_ and ZnO treatments, but the shoot Zn concentration and total Zn uptake were much greater with ZnSO_4_ than the ZnO application. Under given experimental conditions, increasing soil N supply improved shoot N concentration; but had little effect on shoot dry matter production. The concentrations of Zn and Fe in shoots were significantly increased by increasing N application. In case of total uptake of Zn and Fe, the positive effect of N nutrition was more pronounced. Although Se soil treatment had significant effect, N application showed no effect on Se concentration and accumulation in maize shoots. The obtained results show that N fertilization is an effective tool in improving the Zn and Fe status of silage maize and contribute to the better-quality feed.

## 1. Introduction

Agronomic biofortification of field crops with micronutrients is one of the main strategies in sustainable production of healthy and nutrient rich food and feed [1]. Human health problems are associated with micronutrient deficiency worldwide, especially in developing countries, but also the productivity of farm animals grown in these countries is negatively affected [2,3,4]. Low amount of phytoavailable micronutrients in cultivated soils and commonly consumed food and feed crops are main reason of the high prevalence of micronutrient deficiencies in humans [5,6]. According to Hill and Shannon (2019) [7], grazing animals are often exposed to high risk of reduced Zn intake because the pastures usually contain inadequate Zn concentration for a proper animal nutrition that is associated with low amount of phytoavailable Zn in soils. Consequently, there is an increasing trend for biofortification of feed crops with Zn to contribute to better Zn nutrition of livestock. Similarly, enrichment of feed crops with Se is of great importance for animal nutrition and health [8]. Selenium and selenoproteins, such as selenomethionine and selenocysteine, play a role in several critical biological functions in human and animal body and prevent development of various important diseases [9,10]. In essence, micronutrient deficiency affects all phases of food and feed production chain, from field to the final consumer.

Thanks to successful impacts of green revolution, farmers have managed to grow more high-yielding cereal crops, leading to the increased feed and food production and decline in cereal prices. However, this trend had a negative side effect, and resulted in dilution in the concentrations of micronutrients in the food and feed and unintentionally enhanced hidden hunger problem [11,12,13].

Zinc, Fe and Se deficiencies in soils are common in both developed and developing countries [5,8,14]. Soils of Western Balkan countries differ greatly in the concentration and availability of micronutrients, such as Zn, Fe and Se, as their availability is affected by soil factors, such as pH, soil organic matter, fertilization application, micronutrient concentration [15]. Manojlovic and Singh (2012) (15) also found that some fodder crop samples contained Zn and Se below the critical deficiency level and dietary requirement for ruminants.

Dairy cattle nutrition is highly affected by Zn, Fe and Se presence in the feed, because these micronutrients have crucial role in different metabolic processes [4,16]. Many different enzymes in animal body either contain Zn or are activated by Zn. Zinc is required for up to 10% of the proteins in biological systems for their functioning and structural stability [17]. Proteins bind Zn tightly with very high affinity, from picomolar to femtomolar range, to maintain their cellular functions and interactions [18]. Zinc is involved in cell replication, hormone production and immune system and electrolyte balance [19]. Iron makes 90% part of proteins, e.g. hemoglobin. A host of biochemical reactions, especially the enzymes of the electron transport chain (cytochromes) are activated by Fe [19].

Selenium, which acts as an antioxidant, makes an integral part of several enzymes. Selenium as selenocysteine (Se-Cys) is incorporated in the active center of at least 25 selenoproteins [8,20]. Analysis of 105 sheep and 160 cow blood samples collected from different Western Balkan countries indicated low Se nutrition in animals, and therefore the need to improve animal feeds with Se to ensure a better Se nutrition of animals was highlighted [21].

Maize (*Zea mays* L.), the most grown field crops worldwide, provide dietary staple food for > 200 million people with about 20% of their calories needs [22,23]. Furthermore, 67% of maize produced globally is utilized, either as grain or as silage, for livestock feed [24].

In Europe Union, green maize production is also increasing, especially as silage crop, and it was grown on more than 6.4 million hectares in the EU-28 in 2019. The area increased by 0.5 million hectares (+10.9%) compared with 2011 [25]. Its production amounted to 248.6 million tons, nearly 48 million tons more than in 2010 (+23.9%) [25]. This significant increase in production of silage maize is mainly because: (i) it extends the area of tolerable climatic conditions for maize growth, (ii) has high biomass yield, (iii) it represents main component of domestic ruminants diet and (iv) recently it is used as source for biogas production in developed countries [26]. Micronutrients play an important role in producing high-quality maize silage with respect to its mineral status and therefore, to improve crop productivity and its nutritive value, adequate micronutrient concentrations are needed [27]. Maize is extremely sensitive to deficiency of Zn and Fe and therefore, farmers have made a regular agronomic practice of using micronutrient fertilizers [24,28].

New agronomic approaches have been developed to improve capacity of maize plants to absorb more Zn from soils, such as localized ammonium sulphate and superphosphate applications [29]. The form of Zn fertilizers has a significant effect on plant growth and Zn accumulation in plants, especially in high pH soils. Previously, Mortvedt (1992) [30] and Gangloff et al. (2002) [31] have highlighted that the Zn fertilizers applied in high pH soils should have at least 50% water solubility to improve growth and Zn concentrations of plants in high pH calcareous soils.

Recent research on foliar and soil application of Zn and Fe showed that agronomic biofortification is efficient in reduction of these micronutrient deficiencies, particularly in wheat and rice, as main staple crops in human nutrition [32,33,34]. In recent years, it has been shown that foliar spray of a mixture of micronutrient solution containing simultaneously Zn, Se, Fe and iodine to wheat and rice grown in different countries greatly increased grain concentrations of Zn, Se and iodine [35,36]. In these studies, the effect of foliar sprayed Fe on grain Fe was not adequately high compared to Zn, Se and iodine. In previous studies, it has been suggested that N nutritional status of plants has an important role in increasing root uptake, shoot transport and grain deposition of Fe as well as Zn [1,37]. It was interesting to notice that increasing rate of Fe application had little effect on grain Fe; but at a given Fe application rate increasing N application increased grain Fe [32]. Kutman et al. (2010) and Erenoglu et al. (2011) [38,39] showed that increasing N application promotes root uptake of Zn and Fe and increases shoot and grain concentration of these nutrients. Literature reports also suggest that, in case of maize, N application positively affects maize shoot and grain micronutrient concentrations to certain extent under different field conditions [40,41,42,43]. Agronomic biofortification, a widely accepted approach in preventing micronutrient deficiency in several food and feed crops, is not a well- known practice in Western Balkan countries [15].

Considering that, maize silage is one of basic feed component for the dairy cattle in many Balkan countries [44], it is important to investigate the effect of N fertilization on uptake and concentration of Zn, Fe and Se in maize plant. Since N, fertilization is shown to stimulate the uptake and concentration of Zn and Fe and perhaps Se in plants, this may help to produce silage of higher nutritional value, with respect to daily needs of dairy cattle. Practically this fortified silage could lead to reduce the use of different micronutrients supplements.

The present study was planned to examine the effect of N and Zn application on: (1) maize shoot dry matter yield; (2) Zn, Fe and Se concentration and uptake in maize; and (3) the relationship between the concentration of N and that of Zn and Fe in maize plants. Furthermore, the effect of the Zn form applied as a ZnO or ZnSO_4_ on shoot growth, shoot Zn concentration and total shoot Zn uptake was also investigated.

## 2. Materials and Methods

### 2.1. Greenhouse Experiment

A climate-controlled greenhouse experiment was conducted at the Sabanci University campus (40°53′24.5″ N; 029°22′46.7″ E) Turkey.

The soil used for this experiment originated from the Zn-deficient region of Central Anatolia and has clay loam texture with pH 7.6 (H_2_O), 1.5% organic matter, and 18% CaCO_3_. The diethylentriamine pentacetic acid (DTPA)-extractable Zn and Fe concentration was 0.1 mg kg^−1^ and 2.1 mg kg^−1^ soil, respectively, and 0.1 M KH_2_PO_4_ extractable Se was 0.002 mg kg^−1^ soil.

Two experiments were established. In the first one, the experiment consisted of two levels of each N and Zn. The levels for N were 125 and 250 mg N kg^−1^ soil and that of Zn were 1 and 5 mg Zn kg^−1^ soil. The sources of N and Zn were Ca (NO_3_)_2_ 4H_2_O and ZnSO_4_ 7H_2_O, respectively. Similarly, there were two levels of Fe, i.e., 0 and 10 mg Fe kg^−1^ soil and two levels of Se, i.e., 0 and 0.02 mg kg^−1^. Iron and Se fertilizers were applied in form of Fe-sequestrene and Na_2_SeO_4_, respectively.

Basic fertilizers added were phosphorus (P) 100 mg P kg^−1^ soil in the form of KH_2_PO_4_, sulfur (S) 50 mg S kg^−1^ in the form of K_2_SO_4_, and potassium (K) in the form of KH_2_PO_4_ and K_2_SO_4_. All these nutrients and basic fertilizers were homogeneously mixed with 3 kg soil prior to putting the mixture into plastic pots. The experiment was laid out as factorial randomized design with 4 replicates. Twelve seeds of maize (*Zea mays* L.cv. Shemal) were sown in each plastic pot. Shortly after emergence, number of plants was reduced to 6 plants/pot. The pots were irrigated daily with deionized water. When plants were25 days old, their shoot parts were harvested.

In the 2nd experiment, two different forms of Zn (i.e., ZnSO_4_ and ZnO) were used to study how these Zn sources affect the shoot growth and shoot concentration of Zn of maize plants. Zinc was applied at the rates of 0, 0.5, 1, 2.5 and 7.5 mg kg^−1^ soil in the forms of ZnO and ZnSO_4_ at the sowing time. When the plants were 35 days old, plants were harvested and analyzed for shoot production and shoot Zn concentration.

### 2.2. Plant Sampling and Chemical Analysis

Plant shoot materials, after washing with deionized water, were dried at 70 °C for the dry mater determination of shoot weight and analysis of micronutrients. Dried and ground plant samples (0.2 g) were digested with acid [a mixture containing 2 mL of 30% (v/v) H_2_O_2_ and 5 mL of 65% (v/v) HNO_3_] in a closed-vessel microwave system (Mars Express; CEM Corp., Matthews, NC, USA). The digested solution was diluted with DI water. For the determination of Zn, Fe and Se in the digested solution, inductively coupled plasma optical emission spectrometry (ICP-OES) (Vista-Pro Axial, Varian Pty Ltd., Mulgrave, Australia) was used. A dry combustion method (950 °C) using a LECO Tru-Spec C/N Analyzer (Leco Corp., St Joseph, MI, USA) was used for the determination of N concentration in the samples. The certified standard reference materials, obtained from the National Institute of Standards and Technology (Gaithersburg, MD, USA), were used for checking the precision in mineral nutrient analysis.

As standard sample, the SRM 1547 peach leaves, were used and the deviation was below 2%. The total uptake per plant was calculated by multiplying the concentration with dry mater yield. 

### 2.3. Statistical Analysis

The R commander program was used for statistical analysis of the results obtained. Analysis of variance (ANOVA) was used to assess the significance level of the effects of treatments and their interactions on the reported traits. Tukey test at 5% level (P ≤ 0.05) was used for significant difference among means, whenever ANOVA (general linear model) indicated significant effect of treatments. The relationship between the treatments was assessed by linear regression model.

## 3. Results

**Figure 1:**
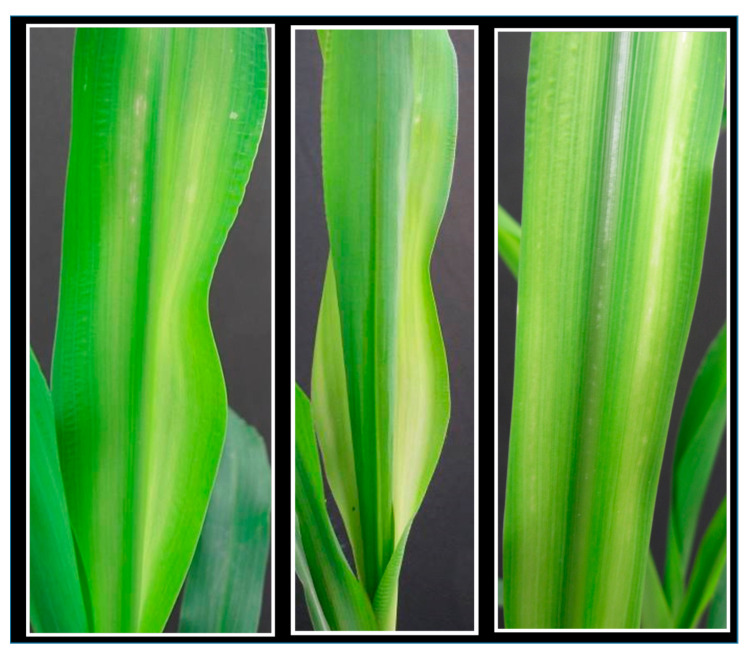
Zinc deficiency symptoms on young leaves of maize plants.

Zinc deficiency symptoms (i.e., development of yellow or yellowish–white stripes along the midrib of younger leaves) appeared in plants grown under low supply of Zn (Figure 1). The symptoms started to develop first following 2 weeks of growth without Zn application under given conditions. Expression of these symptoms was more severe at low N than at adequate N supply. In case of Fe deficiency, uniform chlorosis on younger leaves appeared. Under low N supply, older leaves turn uniformly pale green and then whole shoot look slightly yellowish. The effect of soil N and Zn applications on maize shoot dry matter (DM) yield, shoot Zn and Fe concentrations, and their total uptake per plant was significant.

By contrast, soil N and Zn applications did not significantly affect shoot Se concentration and total Se uptake (Table 1).

Variation in N supply did not show a significant impact on the shoot dry matter yield of the plants at both low and adequate Zn treatment (Table 2), indicating that low N supply was still enough at this growth phase of maize under given experimental conditions.

On the other hand, variation in Zn soil application both under low and adequate N supply significantly affected shoot dry mater production (Table 2). Table 2 shows that the shoot dry matter of plants at low Zn and adequate N supply was 10% higher than the shoot dry matter of plants produced under low Zn and low N treatments. The shoot dry matter of plants under adequate Zn and N supply was 12% higher than those plants grown under adequate Zn but low N supply.

Both Zn concentration and total Zn uptake by shoots were increased by increasing soil Zn application (Table 3). At the low Zn application rate, the plants grown under adequate N supply showed significantly higher concentration and shoot uptake of Zn than the plants grown under low N supply. The increased soil N supply showed positive effect on the shoot Zn concentration and uptake in plants grown with adequate Zn soil application (Table 3).

Selenium soil treatment had significant effect on Se concentration and shoot Se uptake in maize shoot (Table 4), while both N and Zn application did not affect plant Se status (Table 3). Although, not statistically significant, a decrease of the Se concentration could be observed with increase of both N and Zn soil application (Table 3).

**Figure 2:**
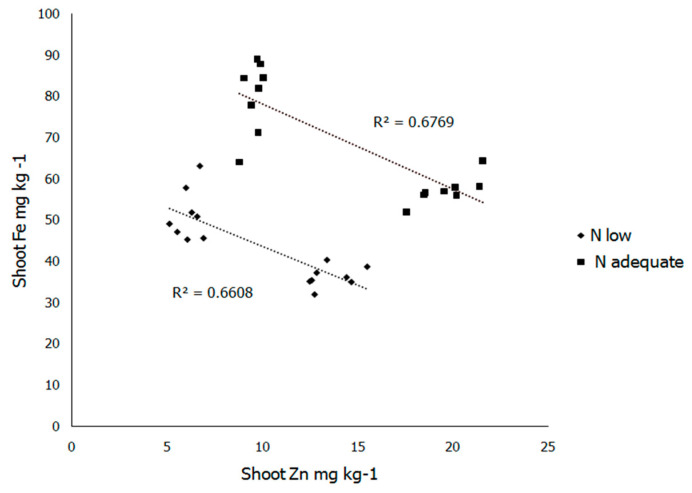
Correlation between shoot Zn and Fe concentrations under different N soil supply. Data for 25. days old maize plants. N rates: low (125 mg of N/kg of soil), (---, ■), and adequate (250 mg of N/kg of soil), (---, ♦).

Shoot Fe concentration and uptake were greatly affected by N and Zn supply (Table 3). Both Zn and Fe uptake were positively affected by increasing soil N supply (Table 3 and Table 4). Shoot Fe concentration and uptake at adequate N application were significantly higher compared to low N application, while the shoot Fe concentration and uptake at adequate Zn supply significantly decreased compared to the low Zn treatment (Table 3). Although increase in soil N supply promoted the Zn and Fe concentration in maize shoot, the ratio between Zn and Fe was mainly affected by the level of soil Zn application (Figure 2).

**Figure 3:**
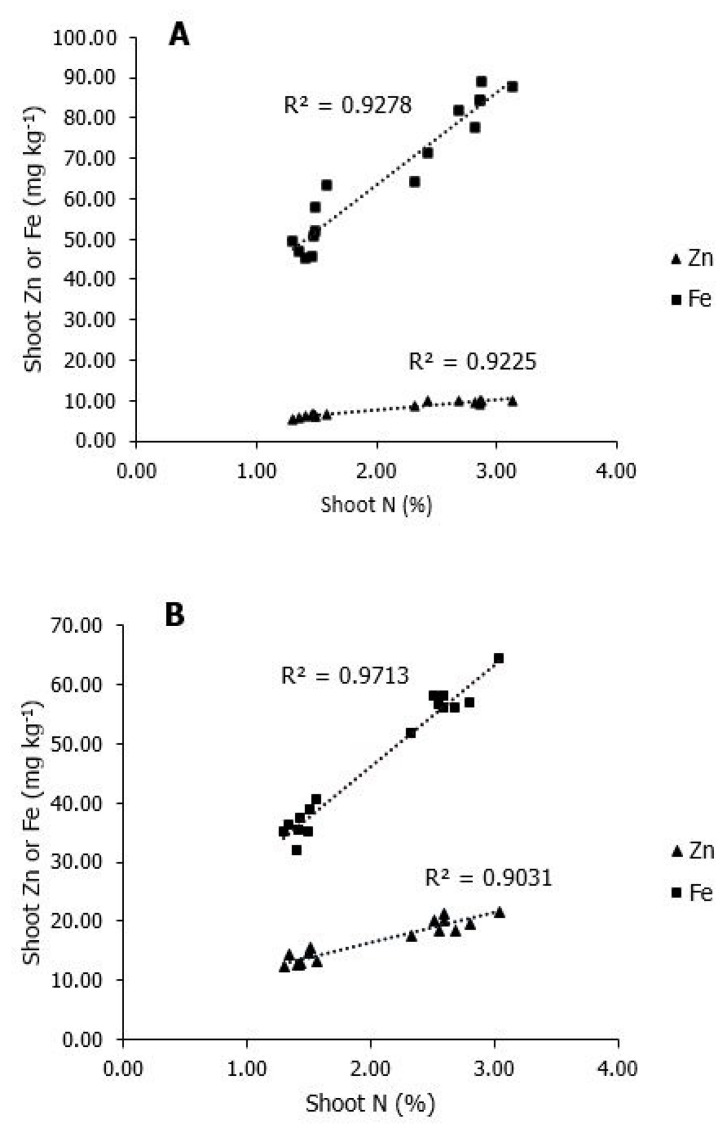
Correlations between Zn, Fe and N concentrations in maize shoot under different Zn soil supply. (**A**) Low Zn soil supply. (**B**) Adequate Zn soil supply. Data for 25 days old maize plants. Fe maize shoot concentration (---, ■), Zn maize shoot concentration (---, ▲).

The relationship between N-Zn and N-Fe concentrations in shoot is shown in Figure 3. The positive impact of N nutrition on Zn and Fe concentrations in shoot in relation to N shoot concentration is shown both under low and adequate Zn supply.

The relation between N-Zn in shoot is highly dependent on availability of N in the soil irrespective of Zn soil availability. Although Zn application decreased shoot Fe concentration (Figure 2), still increase in N supply significantly affected N-Fe relationship, and as in case of N-Zn relationship, N-Fe relationship is strengthened by the increase of N soil availability (Figure 3).

**Figure 4:**
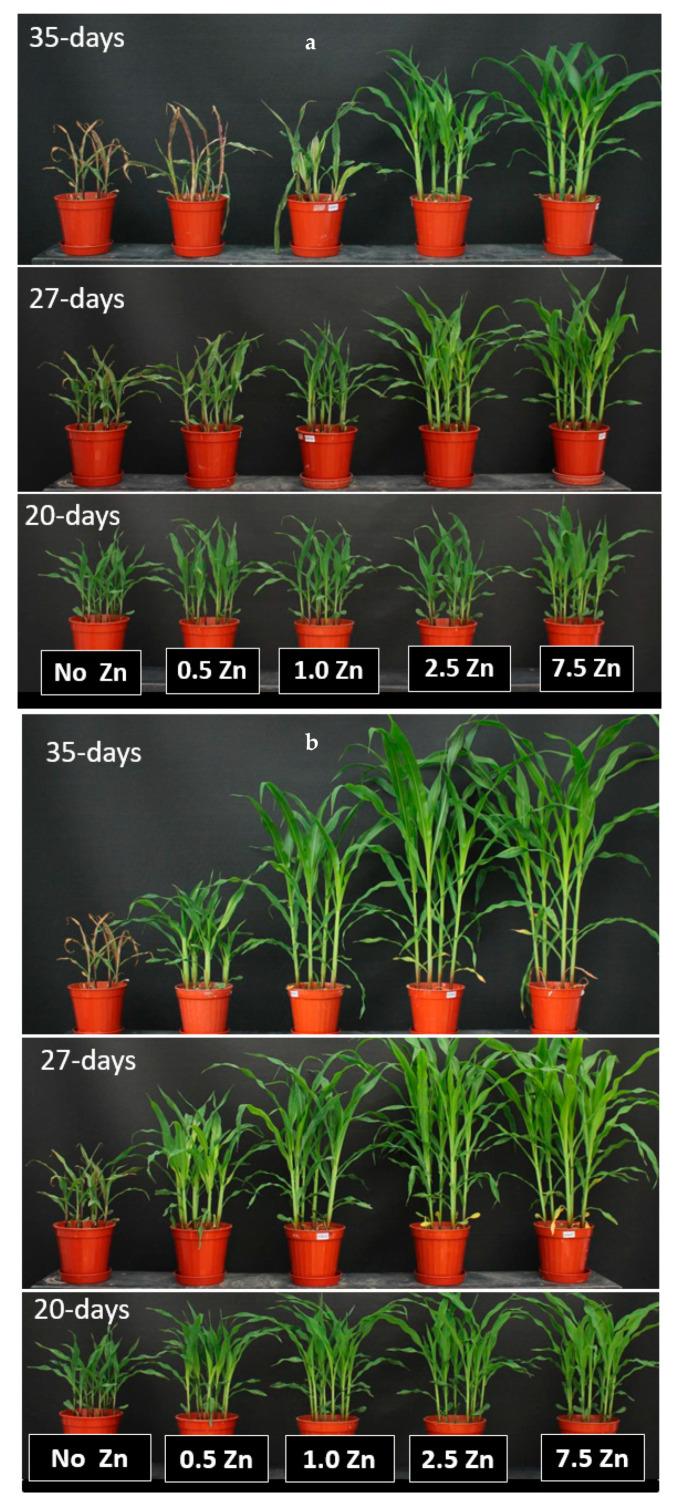
(**a**) Effects of increasing soil Zn supply **in form of ZnO** on growth of maize plants on a Zn-deficient calcareous for 20 days (bottom), 27 days (middle) and 35 days (top). (**b**) Effects of increasing soil Zn supply **in form of ZnSO_4_** on growth of maize plants on a Zn-deficient calcareous for 20 days (bottom), 27 days (middle) and 35 days (top). Zinc has been applied at the rates of 0, 0.5, 1, 2.5 and 7.5 mg Zn kg^−1^ soil in form of ZnO and ZnSO4.

The well-documented effects of Zn nutrition on growth of plants and shoot Zn concentrations and uptake were more distinct with the soil ZnSO_4_ application when compared to the soil ZnO application (Table 5). A progressive increase in shoot dry matter production was observed with increasing soil Zn application, and this increase was stronger and more pronounced in case of soil ZnSO4 application. These much better effects of ZnSO_4_ application on shoot growth than the ZnO application are also shown in Figure 4a,b.

The plants receiving ZnO could not develop well when sol Zn application was <1 mg Zn kg^−1^ soil. However, shoot Zn concentrations showed a clear increase by increasing soil Zn application both with ZnSO_4_ and ZnO treatments. Since the enhancements in shoot dry matter by increasing soil Zn supply was more obvious by ZnSO_4_ application, the shoot total Zn uptake was accordingly much greater with ZnSO_4_ than the ZnO applications (Table 5).

## 4. Discussion

Maize is known to be highly sensitive to low Zn in soils [24]. In good agreement with this, decreases in Zn soil application showed detrimental effects on the shoot growth of maize plants (Table 2, Table 3, Table 4 and Table 5; Figure 4a,b). Very positive response of maize plants to increasing soil Zn application was pronounced in case of ZnSO_4_ application. This indicates clearly that use of water-soluble Zn fertilizers in high pH soils is highly desirable. The results presented here are similar those presented for maize plants grown in calcareous soils in the previous studies [45,46]. ZnO is known to be nearly water insoluble (e.g., 0.0016 g per liter) while ZnSO4 shows very high-water solubility (i.e., 580 g per liter) [47]. Use of water-soluble Zn fertilizer is, therefore, highly desirable for high pH soils. At least 40 to 50% of Zn in each granular fertilizer should be water soluble to achieve a positive agronomic impact on plant growth by using Zn-containing compound fertilizers in high pH soils [30,46,48]. Recently, Degryse et al. (2020) [48] emphasized importance and relevance ofwater-soluble Zn in each granular fertilizer and highlighted that water-soluble Zn rather than the total Zn should be considered in the fertilizer labeling regulations.

Studies dealing with the use of nanoparticle ZnO in Zn fertilization of plants is growing with controversial results and debates [49,50]. One critical debate is related to the very poor solubility of Zn existing in nanoparticulated ZnO. Published evidence shows that solubility and diffusion of Zn from a granular fertilizer which is coated by a bulk or nanoparticulated ZnO are not affected from the size of ZnO used [51]. Therefore, use of nanoparticulated ZnO-containing granular fertilizers in high pH soils may have a very minimal agronomic impact on plant growth and plant Zn uptake when compared to use of fertilizers containing higher percentage of water-soluble Zn.

The present study shows that besides the form of the Zn fertilizer used, the N nutritional level of the plants also influences plant Zn concentration. Although varied soil N supply only slightly affected the shoot dry matter production of plants under given experimental conditions, shoot Zn concentration as well as Zn accumulation (i.e., total Zn uptake by shoot) of plants were significantly increased through the increase in N fertilization of plants (Table 2). These results are like those published by LeBlanc et al. (1997) [40], and Xue et al. (2014) [43] for maize and Cakmak et al. (2010b) [52] for wheat grown under field conditions. Positive effects of increasing N fertilization on plant Zn concentrations have been also shown under greenhouse conditions [53].

In a short-term experiment, Erenoglu et al. (2011) [39] showed that Zn transport through root uptake and root to shoot was significantly promoted by N fertilization. An increased N nutrition of plants showed probably positive effects on abundance of Zn-chelating ligands (such as amino acids and amines) and transporter proteins involved in the root uptake and root to shoot transport of Zn in the plants such as nicotianamine, ZIP family proteins and YSL transporters [37,38]. The presented positive effect of higher soil N supply on Zn concentration and uptake is more emphasized under adequate soil Zn application. This result compares well with those found by Kutman et al. (2010) [38] in wheat. According to Kutman et al. (2010) [38] Zn and N are synergistic in their effects on increasing plant Zn concentrations and their levels in growth medium should be at enough levels to achieve the synergistic effect of N on root Zn uptake.

Like Zn, increasing soil N supply also positively affected shoot Fe concentrations, even at much higher level than Zn (Table 3 and Table 4). Losak et al. (2011) [41] in maize and Aciksoz et al. (2011a) [32] in wheat also found the positive effects of N fertilization on plant Fe concentrations. Interestingly, increasing soil Fe fertilization in the form of FeSO_4_ and FeEDTA had no clear effect on shoot and grain Fe concentrations of wheat plants; but at a given Fe dose, increasing N fertilization significantly improved shoot and grain Fe [32]. The N-dependent increases in plant Fe concentrations were ascribed to N-dependent increases in level of Fe-chelating and transporting nitrogenous compounds such nicotianamine and phytosiderohores. Accordingly, it was shown that improving plants N nutritional status increased root release of phytosiderophores from roots [54]. It is known that phytosiderophores play an important role in mobilization of Fe and Zn from sparingly soluble Fe and Zn sources in soils as well as in root uptake and shoot transport of Zn and Fe within plants [55,56,57].

In contrast to Zn and Fe, increasing N fertilization did not affect plant Se concentration (Table 4), which shows that the increasing effect of N fertilization on plant Zn and Fe seems to be specific. Even, an increasing N fertilization has been found to have an inhibitory effect on Se concentrations of vegetables [58]. Similarly, the concentration and uptake of Se in maize plants was not significantly affected by varied Zn supply (Table 1), which could be explained by different root uptake mechanisms for Se and Zn.

Plants are very responsive to soil Se fertilization and show substantial increases in shoot and grain concentrations of Se as shown in field-grown maize plants by Chilimba et al. (2012) [59] in Malawi, Mao et al. (2014) [60] in China and Ngigi et al. (2019) [61] in Kenya. The results presented in Table 4 compare well with those results from the field trials.

Considering the average concentrations of observed micronutrients in experimental maize plants and dietary requirement of the dairy cattle, we can suggest that biofortified maize grown on the deficient soils can meet the dairy cattle requirements to certain extent. Average Se maize shoot DM levels ranged between 0.04–0.88 mg kg^−1^ (Table 4), while the defined selenium requirement is 0.3 mg/kg of dietary dry matter for all categories of dairy cattle [62].

This indicates that maize silage could be very successfully Se biofortified after optimization of the application rates. In case of Zn, required dietary concentration for milking cows is 63 mg kg^−1^ DM, for heifers 31 mg kg^−1^ DM and dry cows 22.8 mg kg−1 DM [63], while the average maize shoot DM values ranged between 7–24 mg kg^−1^. According to this comparison, Zn biofortified silage could meet the requirements of dry cows and heifers but not those of milking cows. Required dietary concentration of Fe for milking cow is 24 mg kg^−1^ DM [63]. Thus, average range of maize Fe shoot concentration of 36–80 mg kg^−1^ DM could easily meet the dietary requirements of all cattle categories.

The present study showed that N nutritional status represents a key factor in biofortification of silage maize with Zn and Fe. It is suggested that an optimum N fertilization of feed and food crops are required to contribute to better human and animal dietary intake of Zn and Fe. The results also highlighted importance of use of water-soluble Zn fertilizers in Zn biofortification of plants grown on high pH soils.

## Figures and Tables

**Table 1:** Analysis of variance (ANOVA) of effects of soil N and Zn applications on shoot dry matter, Zn, Fe and Se concentration and Zn, Fe and Se uptake in 25-days old plants ^a,b^.

Source of Variation (Treatments)	df	Shoot Dry Matter		Shoot Zn Concentration		Shoot Zn Uptake		Shoot Fe Concentration		Shoot Fe Uptake		Shoot Se Concentration		Shoot Se Uptake	
		SS	F Pr	SS	F Pr	SS	F Pr	SS	F Pr	SS	F Pr	SS	F Pr	SS	F Pr
Soil N (A)	1	0.047	0.461	332.3	<0.001	3802.4	<0.001	6088.3	<0.001	67,088	<0.001	0.0463	0.586	0.221	0.703
Soil Zn (B)	1	1.981	<0.001	1539.2	<0.001	19,448.5	<0.001	1838.8	<0.001	6056	0.039	0.0019	0.911	0.192	0.722
A × B	1	0.058	0.749	34.2	0.172	564.7	0.093	41.5	0.576	32	0.878	0.0299	0.661	0.342	0.635
Experimental error	44	3.756		783.6		8472.5		5752.5		59400		6.7965		66.2	

^a^ Data of 25 days old maize (*Zea mays*) plants grown under greenhouse conditions. ^b^ ANOVA test values: df, SS and F Pr.

**Table 2:** Effect of varied N supply on shoot dry matter production of 25-days-old maize grown at low and adequate Zn levels.

N Treatment ^a^	Shoot Dry Matter (g plant^−1^) ^b,c^	
	Low Zn	Adequate Zn
Low	2.99 ± 0.28 Aa	3.27 ± 0.29 Ab
Adequate	3.02 ± 0.12 Aa	3.37 ± 0.41 Ab

^a^ N treatments: low (125 mg of N kg^−1^ of soil) and adequate (250 mg of N kg^−1^ of soil). ^b^ Data for 25 days old maize plants grown at low (1 mg of Zn kg^−1^ of soil) and adequate (5 mg of Zn kg^−1^ of soil) Zn supply on a Zn-deficient soil under greenhouse conditions. ^c^ The average of 4 independent replicates makes the mean values presented and those in column followed by different uppercase letters and in a row followed by different lowercase letters are significantly different by Turkey test at the 5% level.

**Table 3:** Effect of varied N and Zn supply on the shoot concentration and uptake of Zn, Fe and Se in 25-days-old maize plants.

Micronutrient	Zn Treatment ^a^	Shoot Concentration ^c,e^		Shoot Uptake ^d,e^	
		Low N ^b^	Adequate N ^b^	Low N ^b^	Adequate N ^b^
Zn	Low	7.2 ± 0.5 Aa	10.8 ± 0.5 Ab	21.4 ± 2.3 Aa	32.5 ± 2.4 Ab
	Adequate	16.8 ± 1.1 Ba	23.8 ± 1.2 Bb	54.9 ± 4.3 Ba	80.1 ± 11.8 Bb
Fe	Low	51.4 ± 5.5 Aa	80.0 ± 7.3 Ab	153.5 ± 16.1 Aa	241.6 ± 25.3 Ab
	Adequate	36.3 ± 2.5 Ba	57.2 ± 3.0 Bb	118.3 ± 11.8 Ba	192.8 ± 27.1 Bb
Se	Low	0.83 ± 0.06 Aa	0.80 ± 0.12 Aa	2.4 ± 0.30 Aa	2.5 ± 0.32 Aa
	Adequate	0.88 ± 0.03 Aa	0.71 ± 0.07 Aa	2.8 ± 0.25 Aa	2.4 ± 0.37 Aa

^a^ Data for 25 days old maize plants grown at low (1 mg of Zn kg^−1^ of soil) and adequate (5 mg of Zn kg^−1^ of soil) Zn supply on a Zn-deficient soil under greenhouse conditions. ^b^ N treatments: low (125 mg of N kg^−1^ of soil) and adequate (250 mg of N kg^−1^ of soil). ^c^ Concentration measured in mg kg ^−1^ per micronutrient. ^d^ Uptake measured as μg of micronutrient/plant.^e^ The average of 4 independent replicates makes the mean values presented and those in column followed by different uppercase letters and in a row followed by different lowercase letters are significantly different by Turkey test at the 5% level.

**Table 4:** Effect of varied N supply on the shoot concentration and uptake of Fe and Se in 25-days-old maize plants grown under different Fe and Se soil treatments.

Micronutrient	Micronutrient Treatment ^a^	Shoot Concentration ^c,e^		Shoot Uptake ^d,e^	
		Low N ^b^	Adequate N ^b^	Low N ^b^	Adequate N ^b^
Fe	No treatment	29.2 ± 2.8 Aa	47.2 ± 3.7 Ab	92.3 ± 4.4 Aa	147.4 ± 17.9 Ab
	Adequate	42.6 ± 2.5 Ba	71.0 ± 2.9 Bb	137.7 ± 9.6 Ba	222.3 ± 25.6 Bb
Se	No treatment	0.04 ± 0.01 Aa	0.05 ± 0.01 Aa	0.1 ± 0.0 Aa	0.2 ± 0.0 Aa
	Adequate	0.85 ± 0.03 Ba	0.88 ± 0.08 Ba	2.7 ± 0.2 Ba	2.8 ± 0.4 Ba

^a^ Data for 25 days old maize plants grown at different Fe (0 and 10 mg of Fe kg^−1^ of soil) and Se (0 and 0.02 mg of Se kg^−1^ of soil) supply on micronutrient deficient soil under greenhouse conditions. ^b^ N treatments: low (125 mg of N kg^−1^ of soil) and adequate (250 mg of N kg^−1^ of soil). ^c^ Concentration measured in mg kg ^−1^ per micronutrient. ^d^ Uptake measured as μg of micronutrient/plant. ^e^ Values are means of four independent replicates. ^e^ The average of 4 independent replicates makes the mean values presented and those in column followed by different uppercase letters and in a row followed by different lowercase letters are significantly different by Turkey test at the 5% level.

**Table 5:** Effects of increasing soil Zn supply as ZnO and ZnSO_4_ on shoot dry matter production, shoot Zn concentration and total Zn uptake of 34-days-old maize plants grown on a Zn-deficient calcareous soil. Zinc has been applied at the rates of 0, 0.5, 1, 2.5 and 7.5 mg Zn kg^−1^ soil in form of ZnO and ZnSO_4_.

Zn Source	Zn Supply mg/kg	Dry Matter (g plant^−1^)	Shoot Zn (mg kg^−1^)	Shoot Zn Uptake (µg plant^−1^)
Control	No Zn	0.60 ± 0.02	5.65 ± 0.10	3.4 ± 0.1
ZnO	0.5	0.98 ± 0.11	5.01 ± 0.65	4.9 ± 1.1
	1	1.28 ± 0.32	6.32 ± 0.29	8.1 ± 1.9
	2.5	2.52 ± 1.35	8.09 ± 0.36	20.7 ± 11.9
	7.5	4.64 ± 1.97	9.24 ± 1.10	44.5 ± 23.3
ZnSO_4_	0.5	2.21 ± 0.48	6.88 ± 0.77	15.3 ± 4.3
	1	3.97 ± 0.60	7.24 ± 0.54	28.7 ± 4.6
	2.5	6.16 ± 0.24	8.36 ± 0.27	51.5 ± 2.7
	7.5	7.43 ± 0.30	13.55 ± 1.32	99.6 ± 8.6

## Data Availability

The data presented in this study are available on request from the corresponding author.
